# Fermentation Profiles of Wheat Dextrin, Inulin and Partially Hydrolyzed Guar Gum Using an *in Vitro* Digestion Pretreatment and *in Vitro* Batch Fermentation System Model 

**DOI:** 10.3390/nu5051500

**Published:** 2013-05-03

**Authors:** Jackie Noack, Derek Timm, Ashok Hospattankar, Joanne Slavin

**Affiliations:** 1 Department of Food Science and Nutrition, University of Minnesota, 225 Food Science and Nutrition, 1334 Eckles Ave, St. Paul, MN 55108, USA; E-Mails: noack017@umn.edu (J.N.); timm0125@umn.edu (D.T.); 2 Novartis Consumer Health Inc., Parsippany, NJ 07054, USA; E-Mail: ashok.hospattankar@novartis.com

**Keywords:** short-chain fatty acids, *in vitro* fermentation, tolerance, prebiotic, microbiota

## Abstract

This study investigated the fermentation and microbiota profiles of three fibers, wheat dextrin (WD), partially hydrolyzed guar gum (PHGG), and inulin, since little is known about the effects of WD and PHGG on gut microbiota. A treatment of salivary amylase, pepsin, and pancreatin was used to better physiologic digestion. Fibers (0.5 g) were fermented in triplicate including a control group without fiber for 0, 4, 8, 12, and 24 h. Analysis of pH, gas volume, hydrogen and methane gases, and short chain fatty acid (SCFA) concentrations were completed at each time point. Quantitative polymerase chain reaction (qPCR) was used to measure *Bifidobacteria* and *Lactobacillus* CFUs at 24 h. WD produced the least gas during fermentation at 8, 12, and 24 h (*P* < 0.0001), while inulin produced the most by 8 h (*P* < 0.0001). Each fiber reached its lowest pH value at different time points with inulin at 8 h (mean ± SE) (5.94 ± 0.03), PHGG at 12 h (5.98 ± 0.01), and WD at 24 h (6.17 ± 0.03). All fibers had higher total SCFA concentrations compared to the negative control (*P* < 0.05) at 24 h. At 24 h, inulin produced significantly (*P* = 0.0016) more butyrate than WD with PHGG being similar to both. An exploratory microbial analysis (log_10_ CFU/µL) showed WD had CFU for *Bifidobacteria* (6.12) and *Lactobacillus* (7.15) compared with the control (4.92 and 6.35, respectively). Rate of gas production is influenced by fiber source and may affect tolerance *in vivo*. Exploratory microbiota data hint at high levels of *Bifidobacteria* for WD, but require more robust investigation to corroborate these findings.

## 1. Introduction

Although consumption of fiber from whole foods is ideal, supplemental fiber sources may help increase fiber intake and bridge the gap between fiber recommendations and fiber consumption. *In vitro* studies estimate fermentation profiles including short chain fatty acid (SCFA) production, which is difficult to determine *in vivo*. Structure of the carbohydrate and the number and type of bacterial species present are influential factors in determining fermentation. Metabolites of bacterial fermentation include the gases hydrogen, carbon dioxide, and methane, as well as SCFA. Excess gas production from fermentation can decrease tolerance *in vivo* [1]. Hydrogen can be utilized by other microbiota such as methanogens that form benign methane or sulfate reducing bacteria that form hydrogen sulfide, which is harmful to the colonic epithelium [2,3]. Production of SCFA may be beneficial as they are an energy source to the colonic epithelium and may decrease luminal pH, therefore inhibiting growth of potentially pathogenic bacteria. *In vivo* measurement of SCFA production is difficult due to rapid absorption by colonic epithelium [4]. 

The three dietary fibers evaluated in this study were WD, inulin, and partially hydrolyzed guar gum (PHGG). Wheat dextrin (WD) is produced from the partial hydrolysis and polymerization of wheat starch and is composed of glucose units not digested in the small intestine, but fermented by bacteria in the colon. Little is known about wheat dextrin’s effect on prebiotic bacteria *Bifidobacteria* and *Lactobacillus*. 

Inulin is a fructooligosaccharide and is considered a prebiotic fiber that increases *Bifidobacteria* and *Lactobacillus* bacteria [5]. Small amounts of inulin are naturally present in wheat, onion, banana, garlic, and chicory while numerous products now incorporate inulin into their formulations. Despite being prebiotic, inulin is readily fermented in the large intestine and increases flatulence *in vivo* at doses low as of 5–10 g/day [6]. 

PHGG is a dietary fiber composed of galactose and mannose units produced through controlled breakdown of guar gum. PHGG is sold as a dietary fiber supplement and can be easily incorporated into liquid-based products (juices, yogurt, soups, and enteral nutrition products). PHGG increases total bacteria counts and percent *Bifidobacteria* in humans at doses of 21 g/day [7]. 

The objective of the present study was to compare fermentation profiles including pH, gas production, and SCFA production of dietary fiber supplements WD, PHGG, and inulin with or without a digestion pretreatment to see if differences occur using this procedure. A secondary objective was to explore the levels of prebiotic bacteria levels after fermentation of these fibers.

## 2. Methods

### 2.1. Fibers

Fiber used in this study included WD (Beneﬁber^®^, Novartis Consumer Health Inc., Parsippany, NJ, USA), inulin (Oliggo-Fiber^®^ Cargill, Minneapolis, MN, USA), and PHGG (Taiyo Kagaku Co. Ltd., Mies, Japan). WD is a glucose polysaccharide produced by heating wheat starch under controlled conditions of acidity, moisture, time and temperature. It includes a range of smaller molecular weight glucose polymers with an average degree of polymerization (DP) of 12–25, that contains both typical starch glucosidic linkages (α-1,4 and α-1,6) and linkages atypical of starch (α-1,2 and α-1,3) that are not readily broken down by human digestive enzymes. Inulin is composed of a linear chain of β-(2,1) linked fructose units with a terminal glucose and are indigestible to the hydrolytic activity of human digestive enzymes. PHGG is composed of galactose and mannose units where the mannose units form a linear chain with β-(1,4) bonds and the galactose units branch from the mannose linked by α-(1,6) bonds. PHGG has an estimated molecular weight between 200,000 and 300,000 Da with an average molecular weight of 20,000 Da. 

### 2.2. Digestion Pretreatment

The *in vitro* digestion procedure followed developed by Amrein *et al*. with the modification of omitting bovine bile [8]. Briefly, each fiber (30 g) was suspended in 500 mL phosphate buffer (20 mM, with 10 mM NaCl, pH 6.9) at 37 °C under constant agitation of a magnetic stirrer. Human salivary amylase, (0.25 mL α-amylase, 20 mg/mL, 1 mM CaCl_2_; Sigma Aldrich A1031) was added to the suspension and stirred for 15 min. The pH was adjusted to 2.0 ± 0.1 with HCl. Then, 1.25 mL porcine pepsin (1 mg/mL; Sigma Aldrich P7012) with 15.5 mM NaCl = 0.9 g/L was added and stirred for 30 min. After this, the pH was adjusted to 6.9 ± 0.1 with NaOH. Finally, 5 mL of porcine pancreatin was added (17 mg/g substrate = 510 mg, 50 mg/mL solution; Sigma Aldrich P7545) and incubated for 180 min. Samples were transferred to a Spectra/Por^®^ Biotech Cellulose Ester dialysis membrane tubing with a molecular weight cut off of 500–1000 Da (Spectrum^®^ Laboratories, Inc.; Rancho Dominguez, CA, USA). Dialysis was carried out for 24 h with constant water circulation. Residual was removed and freeze dried. Digestion residue was retained and used for *in vitro* fermentation.

### 2.3. Fecal Collection

Fecal samples from three healthy adult volunteers (two male, one female) free from gastrointestinal disease (ages 18–35) were pooled to make the inoculum for the *in vitro* fermentation. Donors were healthy adults, consuming a non-specific western diet, and had not received antibiotics for three months prior to the study. Donors defecated directly into a bag, placed into an anaerobic bag along with an anaerobic sachet to ensure anaerobic conditions, placed immediately on ice, and returned to the laboratory within 1 h. Samples were delivered to the laboratory and briefly placed in the refrigerator (4 °C) until fecal inoculum preparation.

### 2.4. Fermentation Procedure

Fermentation media, 100 mL serum bottles, and caps were autoclaved to ensure a sterile environment. One liter of fermentation media included 1.992 g trypticase peptone, 7 g sodium bicarbonate, 0.8 g ammonium bicarbonate, 1.144 g anhydrous sodium phosphate dibasic, 1.24 g anhydrous potassium phosphate monobasic, 0.48 g magnesium sulfate, 99.44 mg calcium chloride, 63.424 mg manganese chloride, 15.584 mg cobalt chloride, 5.192 mg ferric chloride, 0.096 mg reaszurin, and distilled water [9]. Reaszurin is a redox agent used to confirm anaerobic conditions through color change.

Half gram (0.5 g) of both the digestion residue and untreated samples were placed into sterile serum bottles. Samples were hydrated using 40 mL of trypticase peptone fermentation media for 12 h and placed in a shaking water bath at 4 °C. Two hours prior to inoculation, bottles were heated to 37 °C. The pooled and homogenized fecal samples were combined with phosphate buffer solution (PBS) (2.4 g anhydrous monobasic sodium phosphate, 0.8 g sodium chloride, in 1 L distilled water) in a ratio of 1:6 feces to PBS to make the fecal inoculum. Following this, two parts reducing solution (950 mL of distilled water, 6.25 g of cysteine hydrochloride, 40 mL 1 N NaOH, 6.25 g of sodium sulfide nonahydrate) were added to 15 parts fecal inoculum [9].

Ten mL of fecal inoculum were added to each bottle along with 0.8 mL of Oxyrase^®^ (Oxyrase Inc., Mansfield, OH, USA) to remove oxygen from the environment. Prior to sealing, bottles were flushed with carbon dioxide to displace oxygen. Bottles were sealed using a rubber cap and aluminum crimp and placed in a shaking water bath at 37 °C.

Digested and undigested fibers were fermented in triplicate including a negative control without fiber for 0, 4, 8, 12, and 24 h. At each time-point, fiber treatments were removed from the water bath and measurements were taken of pH, gas volume, hydrogen and methane gas concentrations, while aliquots were taken for measurement of SCFA and preliminary microbial concentrations. Gas volume was measured using a syringe to pierce the rubber cap and released the pressure within the serum bottle and measuring the amount of gas collected. From the collected gas, hydrogen and methane concentrations were measured using 1 mL aliquot diluted with ambient air. Gas was analyzed using a QuinTron BreathTracker™ SC (QuinTron Instrument Co., Milwaukee, WI, USA). Diluted concentrations were calculated using the equation: *AB^c^*, where *A* = the diluted ppm, *B* = the amount of ambient air used to dilute, and *C* = the number of times diluted. The pH was measured with an Orion 350 PerpHecT (Orion Research, Inc., Beverly, MA, USA). Two mL aliquots were taken from the 24 h time point for exploratory microbial comparison. Then, 1 mL of copper sulfate (200 g/L) was added into the serum bottles to inhibit further fermentation. From this, another 2 mL aliquot was removed and frozen at −80 °C until for SCFA analysis. 

### 2.5. SCFA Analysis

SCFA extraction was modified from Schneider *et al.* [10]. Aliquots were thawed at 4 °C and 1.6 mL of distilled water was added, followed by gentle vortexing and addition of 0.4 mL sulfuric acid (50%), 2 μL 2-ethylbutyric acid (internal standard), and 2 mL diethyl either to each tube. Tubes were again gently vortexed and placed in an orbital shaker for 45 min. The samples were then centrifuged at 3000 rpm for 5 min. The supernatant was transferred to a 5 mL test tube and calcium chloride was added to remove any residual water. Next, the supernatant was filtered with a Fisher Brand 13 mm nylon filter with pore sizes of 0.2 mm (Fisher Scientific, St. Louis, MO, USA) using a 1 mL syringe (Sherwood Medical, St. Louis, MO, USA). Samples were then frozen at −80 °C until analyzed by a Hewlett-Packard model 5890 gas chromatograph (Hewlett-Packard, Palo Alto, CA, USA). The column used was a Stabilwax^®^-DA fused silica column (30 m long; 0.52 mm inner diameter; with a film thickness of 1 μm; Restek, Bellefonte, PA, USA). Each sample was injected at 90 °C and held for 2 min and then the oven temperature increased at a rate of 60 °C/min until it reached 120 °C. Helium was used as the carrier gas.

### 2.6. Microbial Analysis

Microbiota were analyzed using quantitative polymerase chain reaction (qPCR) at the University of Illinois as described previously [11]. Briefly, bacterial DNA from a single triplicate sample at 24 h was purified using QIAamp DNA stool mini kits (Qiagen, Valencia, CA, USA) using the repeated bead beating plus column (RBB + C) method. DNA was quantified using a NanoDrop ND-1000 spectrophotometer (NanoDrop Technologies, Wilmington, DE, USA). Bifidobacterium genus and *Lactobacillus* genus were then quantified via qPCR using specific primers. Amplification was performed on a set of triplicate reactions for each bacterial group within each sample. For amplification, 10 µL final volume containing 2× SYBR Green PCR Master Mix (Applied BioSystems, Foster City, CA, USA), 15 pmol of each primer, and 5 ng of template DNA was used. Pure cultures of each bacterium were utilized to create a five-fold dilution series in triplicate from target species. DNA from each serial dilution was amplified along with experimental DNA samples using a Taqman ABI PRISM 7900HT Sequence Detection System (Applied BioSystems, Carlsbad, CA, USA). The colony forming units (CFU) of each standard curve serial dilution was previously determined by plating the Lactobacillus genus on Difco *Lactobacillus* MRS broth (Becton, Dickenson, and Company, Sparks, MD, USA), and the Bifidobacterium genus on Difco Reinforced Clostridial Medium (Becton, Dickenson, and Company, Franklin Lakes, NJ, USA). Cycle threshold (Ct) values were plotted against standard curves for quantification (log_10_ CFU/µL of sample) of the target bacterial DNA from experimental samples. Microbial counts were measured once for each fiber, so there is no estimate of error in the microbial counts. As a result, no statistical tests are possible.

### 2.7. Statistical Analysis

Data analysis was completed using a general linear model procedure using SAS (SAS Institute, Cary, NC, USA). The fixed effect of fiber was tested. Time was considered a random effect. Least square means were used to determine statistical significance (*P* < 0.05) among treatments. Hydrogen and methane were analyzed in the log_10_ scale and then back transformed. No statistical analysis was completed on microbiota results due to the small sample size. 

## 3. Results

The digestion pretreatment had a significant effect on gas volume and pH levels, whereas no differences were seen in concentration of hydrogen, methane, or SCFA with or without a digestion pretreatment (data not shown). The following results pertain to findings among digested fibers since they more closely mimic an *in vivo* system. 

**Figure 1:**
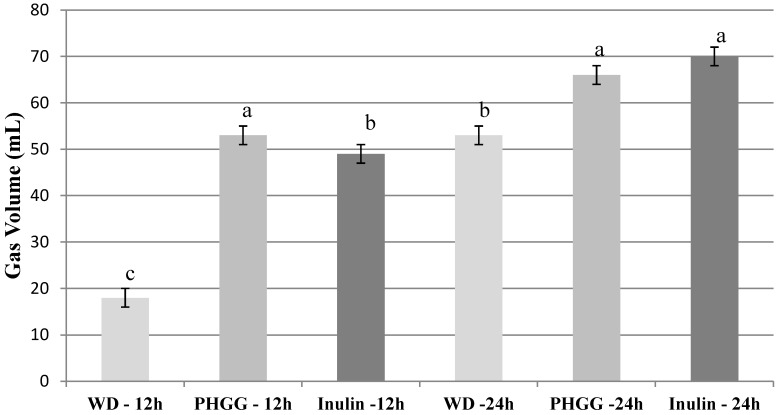
Gas volumes. Values are mean (*n* = 3) ± SE. Within a time point (h), treatments with different letters are significantly different (*P* < 0.05). Gas production at 0 and 4 h was undetectable.

Figure 1 shows gas volume produced during fermentation at different time points. No gas was detectable at 0 and 4 h. Inulin was the only fiber to produce gas at 8 h. Control did not produce detectable gas levels at any time point.

At 12 h, inulin and PHGG had significantly greater hydrogen gas concentration than WD (*P* < 0.05). At 12 h, there was no difference in methane among fibers. At 24 h, inulin had significantly greater hydrogen than WD and PHGG (*P* < 0.0001). At 24 h inulin produced greater methane than WD and PHGG (*P* = 0.01). 

All fiber treatments had significantly lower pH values than the control at every time point. Inulin had the lowest pH values at 4 and 8 h (*P* < 0.0001) (Table 1). At 12 h PHGG had the lowest pH, while inulin had a lower pH than WD (*P* < 0.0001). At 24 h, pH for PHGG was significantly lower than WD (*P* < 0.0001), while inulin was not different from PHGG or WD. 

**Table 1:** pH values *.

Time	WD	PHGG	Inulin	Control	SEM	*P*-value
4 h	6.85c	7.01b	6.60d	7.32a	0.03	<0.0001
8 h	6.62b	6.50c	5.94d	7.16a	0.03	<0.0001
12 h	6.42b	5.98d	6.08c	7.12a	0.02	<0.0001
24 h	6.17b	6.06c	6.11bc	7.06a	0.04	<0.0001

* Within a time point (h) means with different letters are significantly different (*P* < 0.05). Values are reported as mean (*n* = 3).

Table 2 summarizes the production of acetate, propionate, butyrate, and total SCFA. Acetate concentration was greatest for inulin at 8 and 12 h (*P* < 0.0001); however, by 24 h production among the fibers did not differ. All fibers had significantly more acetate than control at 8, 12, and 24 h (*P* < 0.01). 

**Table 2:** Short chain fatty acid (SCFA) concentrations *.

WD		PHGG	Inulin	Control	SEM	*P*-value
Acetate						
4 h	4.6b	2.9c	5.7a	2.0c	0.18	<0.0001
8 h	11.8b	9.4b	17.4a	3.5c	0.81	<0.0001
12 h	14.5b	16.2b	18.1a	5.4c	0.33	<0.0001
24 h	18.6a	19.2a	21.3a	6.9b	1.73	0.0038
Propionate						
4 h	6.0a	4.4b	6.3a	2.2c	0.27	<0.0001
8 h	18.0ab	21.0a	22.4a	11.7b	1.22	0.0027
12 h	23.9b	33.6a	20.9b	13.0c	0.82	<0.0001
24 h	31.0ab	45.1a	23.7b	18.4b	3.17	0.0043
Butyrate						
4 h	2.4ab	1.9bc	2.5a	1.7c	0.09	0.0021
8 h	4.7a	5.1a	4.2a	2.6b	0.25	0.0014
12 h	8.2a	8.2a	5.1b	4.3b	0.34	0.0002
24 h	15.1bc	20.1ab	26.0a	4.9c	2.09	0.0016
Total SCFA						
4 h	12.9a	9.2b	14.5a	5.9c	0.52	<0.0001
8 h	34.5a	35.6a	44.1a	17.8b	2.03	0.0003
12 h	46.5b	58.0a	44.2b	22.7c	1.37	<0.0001
24 h	64.7ab	84.4a	71.0a	30.2b	6.74	0.0064

* Within a row (h), treatments with different letters are significantly different (*P* < 0.05). Values are reported as mean (*n* = 3) (μmol/mL).

At 4 h, inulin and WD produced more propionate than PHGG (*P* < 0.0001). At 8 h, there were no differences in propionate among fibers. PHGG produced significantly more propionate than inulin and WD (*P* < 0.0001) at 12 h. By 24 h, only PHGG had significantly higher propionate concentration than the control. 

At 4 h, inulin had significantly more butyrate than PHGG (*P* = 0.0021), while WD was intermediate between PHGG and inulin. There were no differences in butyrate among fibers at 8 h, while at 12 h WD and PHGG had a significantly greater concentration than inulin (*P* = 0.0002). By 24 h, inulin had significantly more butyrate than WD (*P* = 0.0016), while PHGG was not significantly different from WD or inulin. 

At 4 h, WD and inulin had significantly greater total SCFA than PHGG (*P* < 0.0001). By 8 h, there were no differences in total SCFA among fibers. At 12 h, PHGG had significantly greater total SCFA than WD and inulin (*P* < 0.0001). At 24 h, all fibers had similar total SCFA concentrations. 

At 24 h there were significant differences seen within the proportions of SCFA produced (individual SCFA/ total SCFA). Inulin and WD had a larger proportion of acetate (30% and 29%, respectively) compared to PHGG (23%) (*P* < 0.0001). Propionate proportions were significantly different between all treatments with PHGG (53%) > WD (48%) > inulin (33%) (*P* < 0.0001). Butyrate proportion from inulin (37%) was significantly higher than PHGG (24%) and WD (23%) (*P* < 0.0001). 

No baseline *Bifidobacteria* or *Lactobacillus* measures were taken due to the high cost of the analysis, but after 24 h of fermentation results can been seen in Table 3. No statistical analysis was done due to insufficient sample size.

**Table 3:** Microbial values at 24 h *.

	WD	PHGG	Inulin	Control
*Bifidobacteria*	6.12	5.56	4.54	4.92
*Lactobacillus*	7.15	7.10	6.73	6.35

* No statistical analysis was performed due to small sample size (*n* = 1). Values are log_10_ CFU/µL.

## 4. Discussion

The objective of the present study was to compare fermentation profiles of three soluble and fermentable fibers (WD, PHGG and inulin) after *in vitro* digestion with salivary amylase, porcine pepsin, and porcine pancreatin to predict physiological benefits and tolerance *in vivo.* The digestion step was meant to degrade any digestible carbohydrate or remove sugars used as anti-caking agents added to commercial fiber supplements, which may influence the fermentation profile.

*In vitro* gas production is of interest as discomfort and poor tolerance have been experienced from rapidly fermented fibers *in vivo*. Determinants of discomfort may depend on volume and distribution of gas in the gut [12]. A volume of gas in the proximal colon may be perceived more than the same volume in the distal colon suggesting that rate of fermentation may affect tolerance based on location of fermentation within the colon and slower fermenting fibers may be less likely to cause discomfort than more rapidly fermented fibers [13]. The present study measured gas volume by the overpressure developed in sealed serum bottles by a syringe, which yields a relative volume of gas produced that is useful for comparisons among fibers, but values are not absolute and explains the lack of measureable gas production at 4 h, despite SCFA production. Differences in both volume and rate of gas production among WD, PHGG and inulin were observed in the present study. WD produced significantly less gas than PHGG and inulin at all time points. This suggests WD may be better tolerated *in vivo* than PHGG and inulin. *In vivo* tolerance of WD is maintained with doses up to 45 g/day while excessive flatulence was reported with doses above 50 g/day [14]. High levels of flatulence have been reported after consumption of PHGG at doses of 4 and 21 g/day [15]. Inulin significantly increased flatulence and other adverse effects at relatively lower doses (5–10 g/day) [6]. Therefore, our *in vitro* findings align with the *in vivo* tolerance literature that WD produced less gas and is better tolerated at higher doses than inulin and PHGG. 

Hydrogen and methane are measured during *in vitro* fermentation as an indirect estimate of metabolic pathways utilized by microbiota as colonic bacteria can convert hydrogen into hydrogen sulfide, methane, and acetate [2]. Among these, methane and acetate are preferable products as the presence of hydrogen sulfide is potentially damaging to the colonic epithelium [16]. As hydrogen can be converted into methane, the two have an inverse relationship in the present study between 12 and 24 h where methane increases as hydrogen decreases. Inulin produced significantly greater hydrogen than WD at 12 and 24 h in the present study, which is consistent with previous *in vitro* findings [17]. Differences in concentration ranges compared to a previous *in vitro* study are likely due to disparities in the amount and number of hydrogen producing and methanogenic bacteria present in the donor samples, but we are unable to confirm this since no microbial analysis was done on the donor feces. The pH of the colonic lumen is an indicator of fermentation and also plays a role in the modulation of the gut ecosystem. It has been previously shown that low pH (5.0 and 4.5) promotes the growth of non-pathogenic bacteria such as *Bifidobacteria* (*Bifidobacterium infantis*) and inhibits growth of potentially pathogenic bacteria such as *E. coli* and *C. perfringens in vitro* [5]. All three fibers had pH values significantly lower than the control; however, they were not as low as 5.0–4.5.

There was no difference in total SCFA at 24 h among the fibers and this finding is somewhat expected as the same amount (0.5 g) of each fiber was fermented; however, differences were seen at 4 and 12 h indicating different rates of fiber fermentation. Acetate levels were similar among the fibers at 24 h, although inulin produced more acetate at early time points, which has been seen previously *in vitro* when compared to WD and PHGG [18]. PHGG favored propionate production resulting in significantly more than inulin at 24 h, whereas WD had an intermediate value between the two, which supports previous *in vitro* results where PHGG increased propionate production compared to the control and other fibers [19]. Interestingly, propionate from inulin decreased between 8 and 12 h. Although not well understood, this decrease at 12 h was also found previously with *in vitro* fermentation of inulin and is likely an artifact of this fermentation model [17]. At 24 h inulin produced significantly more butyrate than WD, while PHGG was intermediate to both fibers. This finding is consistent with previous research showing inulin favors butyrate production [18]. Butyrate provides energy for colonic epithelium and can induce apoptosis in cancer cell lines [20]. SCFA tend to be less prevalent in the distal colon due to rapid absorption in the proximal colon. Colonic diseases are often found in the distal colon; therefore, a fiber that is slowly fermented throughout the colon may be beneficial.

Fibers stimulate the growth of *Bifidobacteria* or *Lactobacillus*, which may lead to enhanced regulation of the microbial balance in the colon as *Bifidobacteria* produce SCFA and anti-microbial compounds that may protect against increased growth of potentially pathogenic bacteria such as *E. coli* and *C. perfringens*. A preliminary microbial analysis was conducted on samples after an *in vitro* digestion and fermentation in the present study. Inulin is classified as a prebiotic fiber that selectively stimulates bacteria such as *Bifidobacteria* and *Lactobacillus* [21]. Previous findings of an *in vivo* study that found WD significantly decreased *Clostridum perfringens* with consumption of 8 g/day of WD for 4–5 weeks [22]. A human study found *Bifidobacteria* and *Lactobacillus* increased after consumption of PHGG [7]. Nonetheless, it is important to note results in the present study are preliminary as only one time point of 24 h was examined due to the high cost of microbial analysis and should not be over interpreted. Future studies should include rigorous designs including molecular microbiological techniques to identify microbiota present at baseline and throughout fermentation to identify changes.

Another limitation of this study is that we pooled the three fecal samples to create the inoculum to initiate fermentation. This practice has long been accepted as a standard procedure for *in vitro* fermentations; however a shift toward three separate inoculums may be preferable [23].

## 5. Conclusion

In conclusion, we found differences in the rate of fermentation as demonstrated by differences in gas production and pH values at different time points. PHGG was fermented at an intermediate rate to inulin and WD. Results may indicate better tolerance at higher doses of WD compared to inulin and PHGG and supports *in vivo* studies that found high tolerance of WD. Microbial levels observed at 24 h suggest a possible prebiotic effect of WD reported *in vivo* and warrant further investigation. Overall, *in vitro* batch fermentations provide a useful and economical way of determining estimates of fermentation and microbiota profiles of fibers. 
